# Radiation recall pneumonitis after COVID‐19 vaccination

**DOI:** 10.1111/1759-7714.14239

**Published:** 2021-11-17

**Authors:** Kanako Shinada, Shuji Murakami, Daisaku Yoshida, Haruhiro Saito

**Affiliations:** ^1^ Department of Thoracic Oncology Kanagawa Cancer Center Yokohama Japan; ^2^ Department of Radiation Oncology Kanagawa Cancer Center Yokohama Japan

**Keywords:** COVID‐19 vaccination, non‐small‐cell lung cancer, radiation recall pneumonitis

A 48‐year‐old male was diagnosed with locally advanced, unresectable, non‐small‐cell lung cancer (Figure 1a). He received chemoradiotherapy concurrently with a regimen of cisplatin and docetaxel, and intensity‐modulated radiotherapy (60 Gy in 30 fractions) to the tumor in the middle and lower lobe of the right lung and mediastinum. After the completion of chemoradiotherapy, the anti‐programmed death ligand 1 antibody durvalumab was administered every 2 weeks for a year, in a total of 26 cycles, with no disease progression or severe adverse events.

**FIGURE 1:**
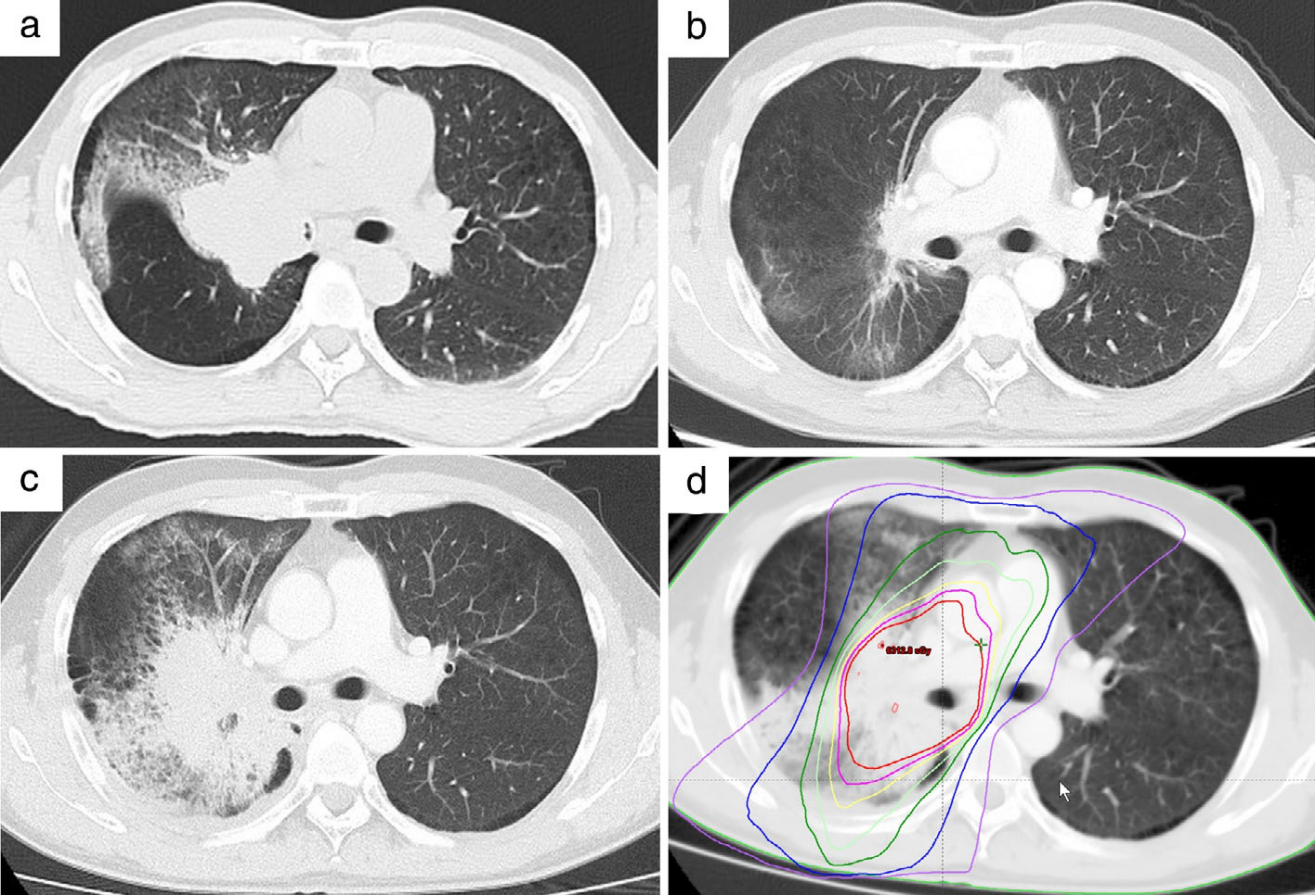
Computed tomography (CT) images of pulmonary opacity during the clinical course of the patient. (a) CT scan at diagnosis revealed a mass lesion in the right lower lobe. (b) There was no evidence of pneumonitis on the CT scan after the completion of durvalumab maintenance treatment following concurrent chemoradiotherapy. (c) Twenty‐one days after the second COVID‐19 vaccination, suspected radiation recall pneumonitis was detected. (d) Comparison of the planned isodose curve in the treatment plan and pneumonitis

The patient received his first dose of a BNT162b2 vaccine against coronavirus disease 2019 (COVID‐19) 8 days after his last dose of durvalumab and the second dose 21 days later. Chest computed tomography (CT) was performed as a routine follow‐up 15 days after the first vaccination and revealed persistent tumor shrinkage and no significant changes relative to previous CT findings (Figure 1b). Nineteen days after the second vaccination, the patient developed fever and a dry cough, and visited our hospital on day 21. CT imaging revealed an infiltration shadow in the right middle and lower lobes (Figure 1c) in an area that overlapped the previous radiation field (Figure 1d).

Radiation pneumonitis usually develops within 6 months after completion of radiation therapy and results in radiation fibrosis in about 1 year. In this case, acute pneumonitis developed in a previously irradiated field after the vaccination, even though a year had elapsed since the last dose of irradiation. We diagnosed the patient with radiation recall pneumonitis. He took prednisolone 0.5 mg/kg body weight/day and recovered quickly.

Radiation recall pneumonitis is defined as acute inflammation within a previously irradiated field after the administration of a triggering agent, such as chemotherapy or an immune‐checkpoint inhibitor. The mechanism of the disease is unclear, but seems to be related to an immune response. The few available reports suggest that this vaccine can induce the radiation recall phenomenon.^1^, ^2^ Cole et al. also reported a case of radiation recall pneumonitis after anti‐COVID vaccination.^3^ In our case, nothing other than the COVID‐19 vaccine was administered after the last CT examination, so the vaccine must have been related to the onset of this case of radiation recall pneumonitis.

## CONFLICT OF INTEREST STATEMENT

Murakami reports the receipt of personal fees from AstraZeneca, Chugai Pharmaceutical, Boehringer Ingelheim, Taiho Pharmaceutical, and Ono Pharmaceutical. Saito reports grants from Chugai Pharmaceutical and AstraZeneca; and personal fees from Ono Pharmaceutical, Nippon Boehringer Ingelheim, MSD, and Novartis Pharma. The other authors report no conflicts of interest.
